# Commercial Day-Old Chicks in Nigeria Are Potential Reservoirs of Colistin- and Tigecycline-Resistant Potentially Pathogenic *Escherichia coli*

**DOI:** 10.3390/antibiotics13111067

**Published:** 2024-11-10

**Authors:** Madubuike Umunna Anyanwu, Nkechi Harriet Ikenna-Ezeh, Simeon Chibuko Okafor, Chinaza Francisca Ezemuoka, Obichukwu Chisom Nwobi, Temitope Mofoluso Ogunniran, Lynda Onyinyechi Obodoechi, Onyinye Josephine Okorie-Kanu, Anthony Christian Mgbeahuruike, Ifeyinwa Riona Okosi, Ishmael Festus Jaja

**Affiliations:** 1 Department of Veterinary Microbiology and Immunology, University of Nigeria, Nsukka 400001, Nigeria; nkechi.ikenna-ezeh@unn.edu.ng (N.H.I.-E.); cezemuoka@gmail.com (C.F.E.); anthony.mgbeahuruike@unn.edu.ng (A.C.M.); 2 Department of Biotechnology and Food Technology, Faculty of Science, University of Johannesburg, Johannesburg 2092, South Africa; 3 Department of Veterinary Pathology, University of Nigeria, Nsukka 400001, Nigeria; simeon.okafor@unn.edu.ng; 4 Department of Veterinary Public Health and Preventive Medicine, University of Nigeria, Nsukka 400001, Nigeria; obichukwu.nwobi@unn.edu.ng (O.C.N.); lynda.majesty-alukagberie@unn.edu.ng (L.O.O.); onyinye.okoro@unn.edu.ng (O.J.O.-K.); 5 Department of Veterinary Medicine, University of Nigeria, Nsukka 400001, Nigeria; temitope.ogunniran@unn.edu.ng; 6 National Veterinary Research Institute, Vom 930001, Nigeria; previfun@yahoo.com; 7 Department of Livestock and Pasture Science, University of Fort Hare, Alice 5700, South Africa

**Keywords:** colistin resistance, day-old chicks, enterobacteria, poultry, tetracycline resistance, tigecycline resistance

## Abstract

**Background:** Frequent use of colistin (COL) and tetracyclines in the Nigerian poultry sector potentially triggers bacterial resistance against COL and tigecycline (TIG), which are last-line antibiotics used to treat multidrug-resistant infections. **Aim/Objectives:** This study aimed to isolate COL- and TIG-resistant *E*. *coli* from commercial day-old chicks distributed to poultry farmers in Nsukka Southeastern Nigeria, assess the production of extended-spectrum β-lactamase (ESBL) and carbapenemase by the isolates, and establish their pathogenic potentials. **Materials and Methods:** Non-duplicate cloacal swabs were systematically collected from 250 randomly selected day-old chicks. MacConkey agar with 1 µg/mL of COL and 16 µg/mL of tetracycline was used for the isolation of putative COL- and tetracycline-resistant *E*. *coli*, respectively. *E*. *coli* isolates were confirmed biochemically using the API20E Gram-negative identification kit and molecularly by polymerase chain reaction targeting the *uidA* gene. Phenotypic COL resistance was established using COL agar and COL disc elution tests, while TIG insusceptibility was determined with disc diffusion. ESBL and carbapenemase production was assessed by double-disc synergy and modified carbapenem inactivation methods, respectively. Pathogenic potentials were determined using phenotypic methods. **Results:** COL- and TIG-resistant *E*. *coli* was recovered from 95 (38.0%) and 62 (24.8%) swabs from the 250 chicks, respectively. None of the isolates were potential ESBL or carbapenemase producers. The COL-resistant isolates displayed pathogenic potentials such as biofilm formation, haemagglutination, cell surface hydrophobicity, surface layer, and gelatinase activities at rates of 30.7%, 8.4%, 33.7%, 23.5%, and 17.6%, respectively. Meanwhile, the TIG-resistant isolates exhibited their respective potentials at rates of 47.0%, 21.0%, 35.5%, 58.1%, and 43.6%. Red, dry, and rough (RDAR) was the predominant curli fimbriae, and the cellulose morphotype portrayed by both the COL- and TIG-unsusceptible potential biofilm-producing isolates. **Conclusions:** This study demonstrates that a significant percentage of commercial day-old chicks distributed to farmers in Nsukka, southeastern Nigeria, are colonized by potentially pathogenic COL- and TIG-resistant *E*. *coli*, which could spread to humans and the environment.

## 1. Introduction

Bacterial antimicrobial resistance (AMR), particularly within the Enterobacteriaceae family, stands as one of the most significant global threats to human and animal health, food security and safety, and the economy. Nigeria’s poultry sector contributes 25% of the country’s consumed animal protein and offers substantial employment opportunities [1,2]. However, the output of this sector faces impediments due to pathogenic and resistant enterobacteria, a consequence of overusing antimicrobial agents for prophylactic and metaphylactic treatment of enterobacterial infections in poultry. Day-old chicks (DOCs) serve as established sources that introduce pathogenic/resistant bacteria into the poultry production chain, even without the use of antibiotics [3,4,5,6]. DOCs are infected pseudo-vertically (horizontal transmission) through contaminated eggshells in the oviduct of the parent stock and/or hatchery and/or through transovarian transmission (vertical transmission) from the ovary of the parent stock [5,6].

COL is a polymyxin antibiotic that is used as a last-resort drug for treating multidrug-resistant (MDR) infections, including infections associated with extended-spectrum β-lactamase (ESBL)-producing and carbapenem-resistant organisms in humans and animals, especially poultry [7,8]. Tigecycline (TIG) is a third-generation tetracycline within the glycylcycline class that is used as a last-line therapy for MDR and extensively drug-resistant (XDR) infections, including carbapenem- and COL-resistant infections in humans [9]. Thus, COL and TIG occupy the highest priority status in critical healthcare scenarios. *Escherichia* (*E*.) *coli*, a highly versatile Gram-negative bacterium of the Enterobacteriaceae family, possesses a distinctive ability to survive and persist in various niches within human and animal hosts, including the gut of DOCs, and the environment [10,11]. Commensal strains of *E*. *coli* crucially maintain the gut microflora balance of DOCs through exclusive competition [1], while pathogenic strains express various virulence factors (VFs) enabling them to cause a wide range of intestinal and extraintestinal infections and outbreaks, posing a serious threat to public and animal health systems [12]. *E*. *coli* strains possessing and expressing VFs pose a greater threat compared to those lacking these factors. Thus, phenotypic evaluation of VFs is important because the presence of a virulence-associated gene does not necessarily indicate its expression, especially when multiple genes are required for the expression of a VF [13]. Virulence factors of *E*. *coli* include bacterial cell surface (such as curli fimbriae, haemagglutinin, surface layer, biofilm, etc.) and secreted (such as haemolysin, proteases, etc.) virulence factors [14]. The expression of these virulence factors facilitates *E*. *coli*’s colonization and invasion of intestinal and extraintestinal sites of the host [12]. Both commensal and pathogenic strains of *E*. *coli* serve as potential reservoirs for AMR. *E*. *coli*, among other bacteria, exhibits the highest propensity to acquire and transfer COL and TIG resistance [15,16]. COL- and TIG-resistant organisms are considered superbugs because they often demonstrate multidrug to pandrug resistance to virtually all available classes of antibiotics, significantly limiting therapeutic options for carriers [9,17]. Hence, COL- and TIG-resistant organisms are the highest-priority pathogens, requiring increased surveillance.

The use of COL and tetracyclines (first-generation tetracyclines such as tetracycline, chlortetracycline, and second-generation tetracyclines such as doxycycline, minocycline) is established as the most important cause of bacterial development of resistance against COL and TIG, respectively [16,18,19]. Thus, TIG resistance confers complete resistance to first-generation tetracycline [20,21]. Research has demonstrated that various antibiotics, including tetracyclines and COL, are commonly used in poultry across many African nations, particularly Sub-Saharan Africa [22,23]. Due to concerns about losing their birds, poultry farmers in Nigeria use massive amounts of antimicrobial agents, including COL and tetracyclines, for prophylactic control and metaphylactic treatment of bacterial infections in chickens as young as one day old [1,24,25]. Although TIG has never been used in Nigeria’s livestock sector, selective pressure for COL and TIG has been exerted in the country’s poultry sector due to the overuse of tetracycline and COL [7,26,27]. Moreover, the use of non-polymyxin and non-tetracycline antimicrobial agents also triggers bacterial COL and TIG resistance [7,28]. At the farm level in Nigeria, tetracycline- and COL-resistant enterobacteria were isolated from chickens at a considerable rate [24,29,30,31,32], and tetracycline resistance rates of 50–100% have been recorded among enterobacterial isolates from commercial DOCs in Nigeria [1,33], suggesting potential overuse of COL and tetracycline in breeder birds. Thus, commercial DOCs distributed to poultry farmers in Nigeria might be colonized by COL- and/or TIG-resistant *E*. *coli*.

Involvement of a COL- and/or TIG-resistant *E*. *coli* strain in avian colibacillosis can aggravate the welfare (such as respiratory signs, growth retardation, reduced feed intake, and increased mortality) and economic (such as condemnation of infected carcasses at slaughter, increased prophylaxis and treatment cost and mortality) problems associated with avian colibacillosis [34]. COL- and/or TIG-resistant *E*. *coli* might be associated with frequent in-shell chick embryo deaths and challenging-to-treat enterobacterial infections, especially colibacillosis, in young chicks—issues which are often experienced by hatchery and poultry farm workers in Nigeria [1,35]. Day-old chicks colonized by pathogenic COL- and TIG-resistant *E*. *coli* pose public health and food safety concerns because these birds constitute potential reservoirs for the transmission of these bacteria to humans through handling of infected birds, consumption of contaminated poultry products, and/or exposure to contaminated environments [1,10,33]. Unfortunately, in Nigeria, acquisition of these organisms from poultry birds through handling and meat consumption is easy because persons who come in contact with poultry (such as farmers, vendors and butchers) rarely wear protective equipment and practice little or no hand hygiene/sanitation, and poultry meat is often processed unhygienically in an unhygienic slaughterhouse environment in the country. COL-/TIG-resistant organisms in the gut of infected individuals could disseminate the resistance among bacteria, thereby compromising antimicrobial therapy and limiting treatment options for infected individuals. This could lead to prolonged illness or even fatalities [9]. Infected individuals could potentially disseminate these superbugs to other places through local and/or international transmission routes.

Given *E*. *coli*’s colonization of the gut of day-old chicks, it serves as a valuable sentinel for COL and TIG resistance surveillance in poultry, which is crucial for devising effective control strategies to limit resistant infections in poultry, enhance empirical treatment of poultry infections, safeguard public health, ensure food safety by limiting the transmission of superbugs from farm to fork, and preserve the effectiveness of these antibiotics for clinical use. While COL resistance has been assessed in DOCs in various countries [4,10,36,37,38], TIG resistance surveillance in poultry has mainly been applied at the farm level [9,16,39], with DOCs being neglected. However, the potential of DOCs distributed to poultry farmers in Nigeria as a reservoir for COL and TIG resistance remains uninvestigated. Therefore, this study aimed to isolate COL- and TIG-resistant *E*. *coli* from DOCs distributed to poultry farmers in Nsukka southeastern Nigeria, evaluate the potential of the isolates to produce ESBL and carbapenemase, and assess their pathogenic potentials.

## 2. Results

### 2.1. Occurrence of COL- and TIG-Resistant E. coli in DOCs

**Figure 1:**
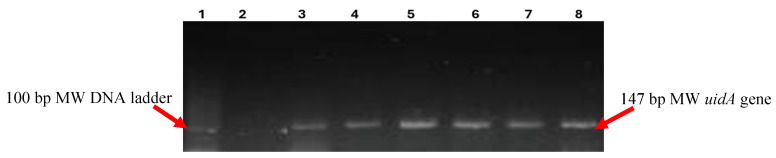
PCR Amplification of the *uidA* gene in colistin- and tigecycline-resistant *E. coli* isolates from commercial day-old chicks in Nigeria. Lane 1 contains the 100 bp molecular weight (MW) DNA ladder, serving as a size reference. Lane 2 represents the negative control, where the PCR mix lacked a DNA template. Lane 3 shows the amplification of the 147 bp *uidA* gene in the positive control strain *E. coli* ATCC^®^ 25922. Lanes 4 to 8 display the amplified 147 bp *uidA* gene in the *E. coli* isolates tested.

Out of the 250 cloacal swabs processed, 95 (38%; 95% CI: 32–44%) grew COL-resistant *E*. *coli*, which was confirmed by the presence of the *uidA* gene (Figure 1), while 62 (24.8%; 95% CI: 19.4 –30.2%) yielded TIG-resistant *E*. *coli* strains, respectively (Table 1). All the COL-resistant isolates had COL MIC ≥4 µg/mL, with the COL agar test being concordant with the COL disc elution test. The 95 COL-resistant and 62 TIG-resistant isolates were, respectively, harboured by 20–93% and 0–48.5% of birds from the hatcheries (Table 1). There was a significant difference (*p* = 0.04) in the number of birds colonized by COL- and TIG-resistant *E*. *coli*. The 95 birds colonized by COL-resistant *E*. *coli* came from all six sampled hatcheries while the 62 birds colonized by TIG-resistant *E*. *coli* came from four of the six hatcheries (Table 1). Twenty-seven (10.8%) of the sampled chicks harboured both COL- and TIG-resistant *E*. *coli*.

### 2.2. ESBL/AmpC and Carbapenemase Production by the Isolates

None of the COL- and TIG-resistant isolates were resistant to cefotaxime and ceftazidime, and none of them showed ESBL/AmpC- and/or carbapenemase production potential.

### 2.3. Pathogenic Potentials of COL- and TIG-Resistant E. coli Isolates

**Figure 2:**
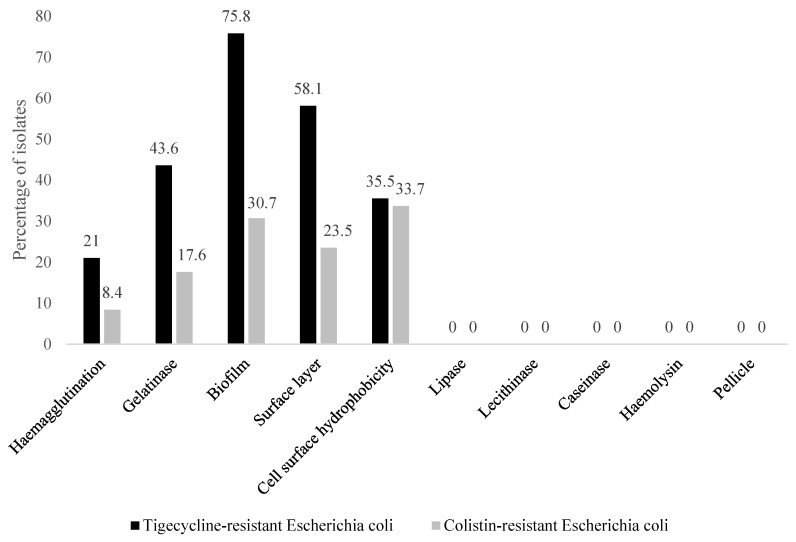
Pathogenic profile of 95 colistin-resistant and 62 tigecycline-resistant *Escherichia coli* isolates from commercial day-old chicks.

**Figure 3:**
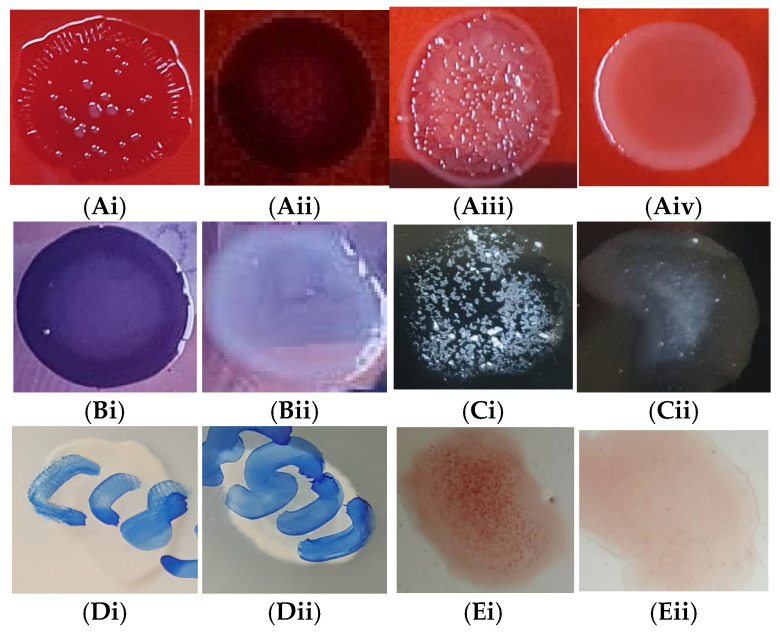
Phenotypic virulence factors expressed by colistin- and tigecycline-resistant *Escherichia coli* isolates from commercial day-old chicks in Nigeria. (**A**) Biofilm (curli fimbriae and cellulose) formation morphotypes assay on Congo red–Coomassie brilliant blue agar; (**Ai**) red, dry and rough (RDAR)—curli and cellulose expression on Congo red–Coomassie brilliant blue agar; (**Aii**) brown, dry and rough (BDAR)—expression of curli but not cellulose; (**Aiii**) pink, dry and rough (PDAR)—expression of cellulose but not curli; (**Aiv**) smooth and white (SAW)—no curli and no cellulose expression. (**B**) Surface layer expression test on Coomassie brilliant blue agar; (**Bi**) bluish morphotype—surface layer-positive on Coomassie brilliant blue agar; (**Bii**) whitish morphotype—surface layer-negative. (**C**) Salt aggregation test with ammonium sulphate salt solution; (**Ci**) salt aggregation-positive—cell surface hydrophobicity; (**Cii**) salt aggregation-negative. (**D**) Gelatin hydrolysis on gelatin agar test; (**Di**) halo around the growth—gelatinase activity-positive; (**Dii**) gelatinase-negative. (**E**) Haemagglutination test with 3% chicken red blood cells; (**Ei**) agglutination/clumping of red blood cells—haemagglutinin-positive; (**Eii**) haemagglutinin-negative.

Of the 95 COL-resistant isolates, 32 (33.7%) expressed cell surface hydrophobicity (Csh), 22 (23.5%) displayed a surface layer (Sfl), 17 (17.6%) showed gelatinase (Glt) activity, 8 (8.4%) exhibited haemagglutination (Hgl), while 29 (30.7%) presented with a biofilm (Bfm), of which 20 (68.7%), 6 (20.7%) and 3 (10.3%) of the isolates showed RDAR, BDAR and PDAR morphotypes, respectively (Figure 2 and Figure 3). The SAW morphotype (non-biofilm-producing) was exhibited by 66 (69.5%) of the COL-resistant isolates (Figure 2). In total, 72 (75.8%) of the 95 COL-resistant isolates displayed potential pathogenicity, whereas 23 (24.2%) of them did not express any of the virulence factors assayed.

Among the 62 TIG-resistant isolates, 13 (21.0%) exhibited Hgl, 27 (43.6%) displayed Glt activity, 36 (58.1%) expressed Sfl, 22 (35.5%) showed Csh, and Bfm 47 (75.8%) of which 30 (63.8%), 11 (23.4%) and 6 (12.8%) showed RDAR, BDAR and PDAR morphotypes, respectively (Figure 2 and Figure 3). Fifteen (24.2%) of the TIG-resistant isolates portrayed the SAW morphotype (Figure 2 and Figure 3). Only 56 (90.3%) of the 62 TIG-resistant isolates expressed pathogenic potential, whereas 6 (9.7%) of them did not express any of the virulence factors assayed.

None of the COL-resistant nor TIG-resistant isolates displayed lipase, lecithinase, and caseinase activity, haemolysis, nor pellicle formation (Figure 2). Not one of them was simultaneously positive for all the tested virulence factors. There was a significant difference (*p* < 0.05) between COL- and TIG-resistant isolates that displayed Hgl, Bfm and Glt activity, and Csh. There was no significant association (*p* > 0.05) between resistance to any of the tested antibiotics and cell surface hydrophobicity. The 56 TIG-resistant and 72 COL-resistant isolates displayed 19 and 14 phenotypic virulence patterns, with Glt-Bfm-Sfl (n = 8) and Csh (n = 22) being the predominant patterns, respectively (Table 2).

## 3. Material and Methods

### 3.1. Ethical Approval

Permission to conduct the study and ethical clearance were obtained from the Institutional Animal Care and Use Committee of the Faculty of Veterinary Medicine, University of Nigeria (protocol code FVM-UNN-IACUC-2023–0145 approved March 2023). Verbal informed consent to inclusion was obtained from all commercial DOC vendors before the sample collection.

### 3.2. Study Area

This study was conducted in Nsukka metropolis Enugu State, southeastern Nigeria. Nsukka is geographically located at coordinates 6°51′24″ N 7°23′45″ E. Poultry is the main animal species slaughtered for animal protein and is consumed by about 483,000 people among the population of Nsukka [40]. Commercial DOC (i.e., newly hatched chicks that have not yet taken water nor feed) vendors in Nsukka source birds from different hatcheries in the northern and, more frequently, the southwestern region of Nigeria [1,33]. The DOCs are transported in boxes from the hatcheries to the study area with a transport time of up to 48 h from southwest and less than 24 h from the north. An average of 10,000 DOCs are distributed to poultry farmers in Nsukka on a weekly basis.

### 3.3. Sample Collection, Bacterial Isolation, and Identification

About 25 min after their arrival from the hatchery and prior to distribution to poultry farmers, we randomly selected 5% of every batch of 200 one-day-old broiler chicks from six hatcheries (identified as A, B, C, D, E, and F, and mostly patronized by DOC vendors) that distributed ≥500 DOCs per week to poultry farmers in Nsukka southeastern Nigeria between April and July 2023. A total of 250 non-duplicate cloacal swabs were collected from selected DOCs on a biweekly basis (to increase the chances of recovering resistant organisms in the event of contamination and/or development of the resistance over time) using cotton swabs. Within 15 min, the swab samples were transported on ice to the Veterinary Microbiology Laboratory, Department of Veterinary Pathology and Microbiology, University of Nigeria, and were processed for COL- and TIG-unsusceptible *E*. *coli*. Each cloacal swab was inoculated in 5 mL of tryptone soy broth and incubated at 35 ± 2 °C for 24 h. A loopful of the broth culture was streaked on tetracycline agar (MacConkey with 16 µg/mL tetracycline (Sigma-Aldrich™)) and colistin agar (MacConkey agar with 1 µg/mL COL (Sigma-Aldrich™)) and incubated at 35 ± 2 °C for 48 h. The minimum inhibitory concentration breakpoints of tetracycline and COL in the media were as recommended by the Clinical Laboratory Standards Institute (CLSI) [41]. Different morphological types were observed and described accordingly. Suspected *E*. *coli* (pinkish/lactose-fermenting, mucoid or non-mucoid, medium-sized, circular, or doughnut-shaped) colonies were selected from each plate and purified via inoculation on fresh tetracycline and colistin agar and incubated at 35 ± 2 °C for 24 to 48 h. Pure cultures of the isolates were morphologically identified by Gram staining to observe Gram-negative medium-sized rods and were sub-cultured on eosin methylene (EMB) agar and incubated at 35 ± 2 °C for 24 h. Isolates with the characteristic greenish metallic sheen appearance on EMB agar were presumptively identified as *E*. *coli* using the Analytical Profile Index API20E Gram-negative rods identification kit (Biomérieux, l’Etoile, France) according to the manufacturer’s directions. *E*. *coli* ATCC 25922 was used as a positive control. Isolates confirmed as *E*. *coli* strains were sub-cultured onto nutrient agar slants, incubated at 35 ± 2 °C for 24 h and stored in refrigerator at 4 °C as stock cultures until needed for further analysis.

#### Molecular Confirmation of *E. coli*

DNA was extracted from presumptive *E*. *coli* isolates using a previously described heat lysis method [42]. Briefly, three colonies from each isolate were transferred into 200 μL of sterile distilled water. The suspension was vortexed, heated at 100 °C for 15 min, and centrifuged at 13,000 rpm for 5 min. The supernatant containing the extracted DNA was then collected into a sterile Eppendorf tube. The quality and purity of DNA extracted from the isolates were evaluated using a NanoDrop Lite 1000 spectrophotometer (Thermo Fisher Scientific, USA). High-quality DNA samples were then stored at −80 °C until the analysis. Molecular confirmation of *E*. *coli* was performed using PCR to target the *uidA* gene, which encodes β-D-glucuronidase [42]. *E*. *coli* ATCC 25922 served as a positive control, while negative controls were prepared by replacing the DNA template with nuclease-free water. The PCR reaction mixture consisted of 12.5 μL of 2× DreamTaq PCR master mix (Thermo Fisher Scientific, SA), 5.5 μL of nuclease-free water, 1 μL of primers targeting the *uidA* gene (forward: AAAACGGCAAGAAAAAGCAG; reverse: ACGCGTGGTTAACAGTCTTGCG), and 5 μL of the DNA template, resulting in a total reaction volume of 25 μL. Thermocycling conditions included an initial denaturation at 94 °C for 2 min, followed by 25 cycles of denaturation at 94 °C for 1 min, annealing at 58 °C for 1 min, and extension at 72 °C for 1 min, with a final extension at 72 °C for 2 min. The PCR products were visualized using standard gel electrophoresis on 2% agarose gels in 0.5× TBE buffer, stained with ethidium bromide (1 mg/mL), and photographed under UV light using a transilluminator (Alliance 4.7, UVltec Cambridge, UK).

### 3.4. Detection of Colistin- and Tigecycline-Unsusceptible E. coli Strains

Phenotypic resistance of the putative COL-resistant isolates to COL was established using COL agar and COL broth disc elution tests in accordance with the CLSI guidelines [41]. *E*. *coli* ATCC^®^ BAA-3170TM was used for quality control for COL resistance.

Phenotypic resistance of the tetracycline-resistant isolates to TIG was determined using disc diffusion screening according to the European Union Committee on Antimicrobial Susceptibility Testing (EUCAST) guidelines [43]. *E*. *coli* ATCC^®^ 25922 was used for quality control for TIG susceptibility.

### 3.5. Detection of Third-Generation Cephalosporin and Carbapenem Resistance, and Production of Extended-Spectrum β-Lactamase/Ampicillinase C and Carbapenemase by COL- and TIG-Unsusceptible Isolates

Resistance of the isolates to cefotaxime (third-generation cephalosporin) and meropenem (carbapenem) were determined using disc diffusion method in accordance with CLSI guidelines [41]. Production of ESBL by the isolates was assessed using a modified version of the double disc synergy test (DDST) as per Kaur et al. [44]. Briefly, a disc (Oxoid, Bangistoke, UK) impregnated with amoxicillin–clavulanic acid (AMC, 20/10 µg) was placed at the centre of Mueller–Hinton agar plate spread-inoculated with colonial suspension of each isolate in tryptone soya broth adjusted to 0.5 McFarland’s turbidity standard equivalent to 1 × 10^8^ colony-forming unit (cfu)/mL. Discs (Oxid, Bangistoke, UK) impregnated with cefotaxime (CTX, 30 µg), ceftazidime (CTZ, 30 µg) and cefepime (CFP, 30 µg) were placed on opposite sides of the amoxicillin–clavulanic acid disc, 1.5 cm apart. The plates were incubated at 35 ± 2 °C for 18 h. Development/extension of the inhibition zone towards the clavulanate disc from CTX, and/or CTZ disc, and/or CFP disc was indicative of a potential ESBL-positive and/or AmpC-positive organism [44]. *E*. *coli* ATCC 25922 was used for quality control for ESBL production.

Carbapenemase activity was assayed using the modified carbapenem inactivation method (mCIM) according to the CLSI guidelines [41] and simplified carbapenem inactivation method (sCIM), as previously described [45]. Briefly, for mCIM, a 1 µL loopful of each isolate was inoculated into 2 mL Brain Heart Infusion and vortexed for 15 s. A 10 µg meropenem disc (Oxoid, Bangistoke, UK) was added to the suspension and incubated in ambient air for 4 h at 35 ± 2 °C, then the disc was placed on a Mueller–Hinton agar (MHA) plate which had previously been inoculated with colonial suspension (0.5 McFarland’s turbidity equivalent to 1 × 10^8^ cfu/mL) of a meropenem-susceptible *E*. *coli* ATCC 25922 indicator strain. The inhibition zone was measured after 18 hours of incubation at 35 ± 2 °C in ambient air. Isolates with an inhibition zone of 6–15 mm or the presence of pinpoint colonies within a 16 to 18 mm zone were regarded as carbapenem-resistant; those with zone ≥19 mm were regarded as carbapenem-susceptible; whereas 16–18 mm or a diameter of ≥19 mm with the presence of pinpoint colonies within the zone was considered indeterminate [41]. For the sCIM test, an imipenem disc (10 µg) was impregnated with each of the isolates; then, the disc was placed upside down on MHA plate previously inoculated with meropenem-susceptible *E*. *coli* ATCC 25922. Plates were incubated at 35 °C for 18 h. An isolate with an inhibition zone of 6–20 mm or colonies within a ≤22 mm zone was considered carbapenem-resistant; isolates with an inhibition zone ≥26 mm were considered carbapenem-susceptible; whereas isolates with a 23–25 mm zone of inhibition of was regarded as indeterminate [45].

### 3.6. Detection of Pathogenic Potentials

#### 3.6.1. Haemolysis

Haemolysin production was assessed using a plate haemolysis test as described by Shah et al. [14]. Briefly, 10 µL of 0.5 McFarland’s turbidity suspension (equivalent to 1 × 10^8^ cfu/mL) of each isolate was spot inoculated on blood agar (tryptic soy agar with 5% *v/v* sheep blood) and incubated at 35 ± 2 °C for 24 h. Any isolate with a clear transparent zone surrounding the growth was recorded as haemolytic [14]. *Pseudomonas aeruginosa* ATCC 27853 was used as a quality control strain for haemolytic activity.

#### 3.6.2. Gelatin Degradation

Gelatin agar (tryptic soy agar with 3% gelatin *w*/*v*) was spotted with 10 µL of 0.5 McFarland’s turbidity suspension of each isolate and incubated at 35 ± 2 °C for 48 h [14,46]. The presence of a transparent halo surrounding the growth following the flooding of the agar plate with Frazier solution (mercuric chloride 15 g, hydrochloric acid 37%, 20 mL distilled water, 100 mL) indicated gelatinase production. *Pseudomonas aeruginosa* ATCC 27853 was used as a quality control strain for gelatinase activity.

#### 3.6.3. Casein Hydrolysis

Casein agar (tryptic soy agar with 5% *w/v* soluble casein) was spotted with 10 µL of 0.5 McFarland’s turbidity suspension of each isolate and incubated at 35 ± 2 °C for 48 h [47,48]. The presence of a clear transparent zone surrounding the growth on flooding the agar plate with Frazier solution portrayed caseinase production. *Pseudomonas aeruginosa* ATCC 27853 was used as a quality control strain for caseinase activity.

#### 3.6.4. Lipid and Ester Hydrolysis

The ability to produce lipases and/or esterases which break down lipids and/or esters was determined according to the previously described protocol [48,49]. Briefly, 10 µL of 0.5 McFarland’s turbidity suspension of each isolate was spotted on tween 80 agar (tryptic soy agar with 1% *v/v* tween 80 and 0.01% phenol red) and tween 20 agar (tryptic soy agar with 1% *v/v* tween 20 and 0.01% *w/v* phenol red dye) and incubated at 35 ± 2 °C for 48 h. An isolate with a yellowish zone surrounding the growth was regarded as a lipase producer [48,49]. *Pseudomonas aeruginosa* strain ATCC 27853 was used as a quality control strain for lipase/esterase production.

#### 3.6.5. Lecithin Hydrolysis

Egg yolk agar (tryptic soy agar with 2.5% *w/v* egg yolk emulsion) was spotted with 10 µL of 0.5 McFarland’s turbidity suspension of each isolate and incubated at 35 ± 2 °C for 24 h [50]. The presence of a clear transparent zone surrounding the growth was considered positive for lecithinase activity [50]. *Pseudomonas aeruginosa* strain ATCC 27853 was used as a quality control strain for lecithinase activity.

#### 3.6.6. Surface-Layer Expression

Surface-layer (S-layer) expression was assessed per Igbinosa and Beshiru [46]. Briefly, 1 µL of 0.5 McFarland’s turbidity suspension of each isolate was spotted on Coomassie brilliant blue agar (tryptic soy agar with 0.1 mg/mL Coomassie brilliant blue R 250 [Merck, Darmstadt Germany]) and incubated at 35 ± 2 °C for 24 h. Isolates whose growth were bluish were considered to be S-layer-producers, whereas those whose growth appeared whitish or creamy were considered to be non-S-layer-producers [46]. *Lactobacillus brevis* ATCC 14869 was used as a quality control strain for S-layer expression.

#### 3.6.7. Biofilm (Curli Fimbriae and Cellulose) Formation on Organic Surfaces

The ability to form biofilm on organic surfaces was determined as previously described [51]. In brief, 1 µL of undiluted overnight culture of each isolate was inoculated on Congo red–Coomassie brilliant blue (CRCBB) agar (tryptic soy agar with 40 µg/mL Congo red dye and 20 µg/mL Coomassie brilliant blue dye). The CRCBB agar plates were incubated at 25 ± 1.0 °C. The plates were visually examined after 48 h for up to 96 h, and the biofilm-producing morphotypes were categorized as RDAR (red, dry and rough), indicating the presence of curli fimbriae (curli) and cellulose; pink, dry and rough (PDAR), indicating the expression of cellulose but not curli; BDAR (brown, dry and rough), indicating expression of curli but not cellulose; and smooth and white (SAW), showing no signs of curli or cellulose (i.e., no biofilm) production [51]. *E. coli* ATCC 25922 was used as a quality control strain for curli expression and cellulose production.

#### 3.6.8. Pellicle Formation

The ability to form biofilm (pellicle) at the air–liquid interface was assessed by a broth pellicle test as described by Dawadi et al. [52]. Briefly, 2 to 3 colonies of each isolate were inoculated in 5 mL of tryptone soya broth. The broth culture was then incubated without shaking (stagnant) at 35 ± 2 °C for 24 h and examined visually. The presence of an aggregative matrix layer (pellicle) on the surface of the broth implied pellicle formation [52]. *E*. *coli* ATCC 35218 was used as quality control strain for pellicle formation.

#### 3.6.9. Cell Surface Hydrophobicity

Cell surface hydrophobicity was determined by a salt aggregation test according to the method described by Farid et al. [53], with slight modification. Briefly, 25 µL of 2 M solution of ammonium sulphate was emulsified on a microscopic slide with 2 to 3 colonies of each isolate. Formation of aggregates (clumping) with salt particles was considered to be a positive indicator of cell surface hydrophobicity [53]. *E*. *coli* ATCC 8099 was used as quality control strain for cell surface hydrophobicity.

#### 3.6.10. Haemagglutination

Expression of haemagglutinin was assayed according to the method described by Hagos et al. [54]. Briefly, isolates were inoculated into tryptic soy broth and incubated at 35 ± 2 °C for 48 h. Then, 25 µL of 3% chicken red blood cells and 25 µL of the broth were dropped onto a white tile, which was rocked for 5 min and then examined visually. An isolate that clumped the red blood cells forming agglutination was considered haemagglutinin-positive [54]. *E*. *coli* ATCC 25922 was used as a quality control strain for haemagglutination.

### 3.7. Data Analysis

The results of the various tests were entered into Microsoft Excel TM (Microsoft, Redmond, WA, USA). Data on the occurrence of COL- and TIG-insusceptibility were exported to SPSS v.15.0 (SPSS, Chicago, IL, USA) and GraphPad Prism statistical package v.8.3.1 (GraphPad Software, La Jolla, CA, USA) for analysis. The frequency, percentage and 95% confidence interval of variables were calculated as appropriate. Fisher’s exact test was used to determine the possible association between variables and the number of birds colonized, and the pathogenic potentials.

## 4. Discussion

The isolation of COL-resistant *E*. *coli* from 38% and TIG-resistant *E*. *coli* from 24.8% of birds in this study indicates that a significant percentage of DOCs distributed to poultry farmers in the Nsukka southeastern Nigeria region constitute potential reservoirs for COL and TIG resistance. This finding is a cause for concern because these birds potentially serve as reservoirs and disseminators (by fecal shedding) of COL- and TIG-resistant organisms into the environment, thereby posing a health threat to individuals (animals and humans) that come into in contact with them. Veterinarians, bird caretakers, poultry bird meat processors and manure users could acquire these organisms following contact with feces from the birds or contact with fomites contaminated by the organisms. Potential buyers who could handle the bird carcasses and consumers of meat from these colonized birds are also at risk of getting infected, especially as poultry meat is processed in unhygienic conditions in Nigeria. The DOCs in this study were possibly infected with *E*. *coli* through transovarian (vertical) transmission from parent stock to the egg from which the DOCs originated [5,55]. Although the usage of antimicrobial agents in the breeder chicken farms and hatcheries were not assessed in this study, the phenotypic COL and TIG resistance observed may be due to selective pressure associated with the overuse of antimicrobial agents, especially COL and tetracycline in the Nigerian poultry sector, as reported [2,7,24,25]. It is also possible that the DOCs were exposed to antibiotics such as COL and tetracycline in the hatcheries, as studies showed that antimicrobial agents are sometimes administered in ovo or by subcutaneous injections together with Marek’s disease vaccination to hatchlings to control early mortality rates associated with colibacillosis [1,56]. TIG is a third-generation tetracycline within the glycylcycline class, whose resistance in bacteria is mainly caused by selective pressure following the use of tetracycline and TIG [9]. TIG has never been used in livestock in Nigeria, but tetracycline and COL are typically administered to poultry bird flocks in drinking water during the brooding period (1 to 4 weeks) with a prophylactic purpose and metaphylactic treatment of poultry enterobacterial infections [7,24,31]. There is also a possibility that COL and/or tetracycline were in the feed consumed by the breeder birds, as the use of these antibiotics as growth-promoters has been reported in Nigeria [24,57]. Therefore, COL and TIG resistance observed in this study may be due to the acquisition of genes encoding COL and TIG resistance, and/or other intrinsic COL and TIG resistance mechanisms [9,16]. The dominance of COL-resistant isolates in our study may possibly be due to the overuse of COL rather than tetracycline in breeder stock/hatcheries in Nigeria. However, the findings of this current study call for attention to the use of antimicrobial agents (especially colistin and tetracycline) in poultry, especially in breeder farms in Nigeria, and the potential of DOCs serving as a source of COL- and TIG-resistant enterobacteria, which are highly resistant organisms that can jeopardize antimicrobial therapy and are often associated with difficult-to-treat infections in infected carriers. Unfortunately, there are limited therapeutic options for infections associated with COL and TIG resistance. Moreover, DOCs could serve as a source of COL and TIG resistance in poultry farms in which polymyxins and tetracyclines or any other antimicrobial agent have never been utilized. Lamentably, the presence of COL- and TIG-resistant *E*. *coli* in the gut of DOCs could result in the widespread transmission of the organisms, especially if antimicrobials are used in raising colonized chicks [58].

In this study, varying percentages of birds from all the sampled hatcheries harboured COL- and TIG-resistant *E*. *coli*, suggesting that these hatcheries were possibly contaminated by these organisms. This finding also suggests that the DOCs may also have been colonized by the organisms through pseudo-vertical transmission from contaminated eggshells. Cross-contamination of eggs and hatchlings (through the hands of hatchery workers and surfaces of egg trays, dust, etc.) in hatcheries has been reported as a potential source for the horizontal transmission of bacteria to hatchlings [59]. This is plausible, since COL and TIG resistance have been widely reported in enterobacteria isolated from human communities in Nigeria [8,60]. Moreover, studies have reported that chicks hatch with a sterile intestinal tract, but the microbial populations of DOCs (chicks that have neither eaten nor been placed in a production farm) depend on bacteria ingestion from hatching debris and the environment [1]. Since the birds in this experiment were sampled while still in transport boxes, environmental contamination could have occurred through interaction with dust, feces, and/or the cartons during transport [10,59]. Notably, hatcheries and DOCs in Nigeria have been found to be contaminated/infected by a diverse range of resistant bacteria, including *E*. *coli* [1,33,61]. Nonetheless, the isolation of these organisms forms a considerably high percentage (20–93% and 0–48.53% for COL- and TIG-resistant strains) of birds from the hatcheries suggests potential concerns about the transmission and persistence of COL and TIG resistance within the specific hatchery environment.

The 38% COL-resistant *E*. *coli* isolation prevalence in this study is higher than the 36.1% COL-resistant *E*. *coli* prevalence among DOCs reported in Vietnam [62]. Ahmed et al. [37] reported a 6% COL resistance prevalence among farms holding DOCs in Bangladesh. Coppola et al. [4] recovered COL-unsusceptible *Enterobacter cloacae* from some DOCs imported from Brazil into Uruguay. But our results contrast with the findings of Dougnon et al. [10] and Osman et al. [38], who did not detect COL resistance among *E*. *coli* isolates from DOCs imported into Benin Republic and DOCs in Egypt, respectively. The variation in the results of the studies is probably due to differences in sampling methods, the type of sample analyzed, the method (whether phenotypic or molecular) used to detect COL and/or TIG resistance, the degree of contamination and antimicrobial usage of hatcheries/DOCs, handling/transportation of DOCs, and antimicrobial usage and management in the parent stock farms in the study areas. Notably, a COL resistance rate of 5.7% was detected among enterobacterial isolates from adult chickens administered antimicrobial agents, including colistin, in southeastern Nigeria [24]. Although TIG resistance in DOCs has not been investigated, leading to lack of data with which to compare our results, reports from Egypt [63], China [64,65] and Pakistan [39,66] showed that adult chickens harboured TIG-resistant *E*. *coli*. Interestingly, Chinese investigators isolated TIG-resistant *E*. *coli* from chicken eggs [67], which could possibly be source for colonization of DOCs by the organism. Because expression of a trait could be hindered, we assayed the isolates for ESBL and carbapenemase irrespective of whether they exhibited resistance to third-generation cephalosporin and/or carbapenem. It is encouraging that neither third-generation cephalosporin resistance/ESBL production was observed in this study. This suggests that the parent stocks possibly were not exposed to extended-spectrum cephalosporin (ESC) antibiotics. However, in Nigeria, resistance to third-generation cephalosporins and ESBL production has been reported among enterobacterial isolates from DOCS [1,33] as well as adult chickens [68]. Elsewhere, ESC resistance/ESBLs has also been detected among enterobacterial isolates from DOCs [69,70,71]. Similarly, neither carbapenem resistance nor carbapenemase production was detected in this study, despite using two carbapenemase detection methods that have been adjudged to detect all types of carbapenemase, including the rare types [45]. This is similar to the findings reported by Dougnon et al. [10], who also did not detect carbapenem resistance among *E*. *coli* isolates from DOCs. Nevertheless, Jibril et al. [33] observed meropenem (a carbapenem) resistance among *Salmonella* isolates from DOCs in Nigeria. Although the antimicrobial use in the parent stock farms was not assessed, carbapenems and ESCs are not known to be used in the poultry sector in Nigeria. However, the use of ceftiofur (a cephalosporin) and other β-lactam antibiotics such as ampicillin and amoxicillin in the sector has been reported [2,25,72]. These antibiotics can trigger resistance to ESCs and carbapenems.

*E*. *coli* is one of the principal causes of early chick mortality, accounting for 50% of total flock loss, adult bird morbidity, and mortality, leading to a decline in production and economic revenue in the poultry industry [55]. It is also one of the most common zoonotic organisms causing hospital- and community-acquired infections, including urinary tract infections (UTIs), which affect more than 150 million people annually worldwide [73]. Therefore, the expression of VFs by *E*. *coli* is a major One Health concern. Phenotypic studies can suggest the potential pathogenicity of an *E*. *coli* strain. In this study, the majority of the COL-resistant (75.8%) and TIG-resistant (90.3%) isolates are potentially pathogenic, having expressed one or more of the tested pathogenic potentials. Regarding *E*. *coli*, virulence results from the cumulative impact of one or several VFs, distinguishing potential pathogenic strains from harmless intestinal strains [74]. A considerable percentage of isolates (8.4% and 21.4% for COL-resistant and TIG-resistant strains) in this study exhibited haemagglutination, which is an attack on erythrocytes mediated by cell surface haemagglutinin. Haemagglutinin enables *E*. *coli*’s colonization in intestinal and extraintestinal infections, helping the organism adhere to the epithelial cells of the intestines, kidneys, and lower urinary tract, and to stimulate cytokine production by T cells [75]. Haemagglutinin is regarded as a hallmark virulence factor expressed by Urogenital Pathogenic *E*. *coli* (UPEC), since it enables adhesion to lower urinary tract epithelial cells, which is crucial to the organism’s resistance to the host immune response and its ability to ascend to the upper parts of the urinary tract [54]. Therefore, it is of public health concern because individuals that come into direct or indirect contact with colonized DOCs could acquire these organisms. Although the type of haemagglutinin expressed by the isolates was not assessed in this study, it is worth noting that haemagglutinating activities can be categorized into two types—mannose-resistant haemagglutination, in which mostly P fimbria is expressed and which usually seen in isolates from UTIs; and mannose-sensitive haemagglutination, wherein type 1 pili/fimbriae is expressed and is found mostly in normal gut microbiota [54]. Interestingly, none of the isolates in this study were haemolytic, corroborating the findings of Hogan et al. [76], who suggested that haemolysis does not correlate with hemagglutination.

Gelatinase is a protease that degrades collagen, laminin, gelatin, numerous proteins/peptides, and so on, thereby helping the organisms invade the host. It is one of the virulence factors of *E*. *coli* responsible for pathogenicity in different diseases, particularly UTIs, as it has been associated with adherence and biofilm formation [14]. Therefore, the expression of gelatinase by a sizeable percentage of isolates (43.6% and 17.6% of the TIG- and COL-resistant strains) in this study is of public health concern, as these organisms could potentially cause difficult-to-treat infections in infected carriers. Biofilm is one of the important virulence factors of *E*. *coli*, consisting of bacterial populations that are adherent to each other and/or surfaces or interfaces and enclosed in a self-produced matrix that is often made up of proteins, polysaccharides, and/or extracellular DNA [51]. Agar plates containing Congo red and Coomassie brilliant blue dyes are used to observe curli (aggregative amyloid fibres) and cellulose, which determine the ability of different *E*. *coli* strains to produce biofilm [51,77]. Curli and cellulose also determine the complex macroscopic architecture of the biofilm, contribute to adhesion to surfaces and cell aggregation, and might also play a role in resistance to environmental stressors such as desiccation and disinfection [49]. In our experience, a considerably moderate-to-high percentage (35.7% and 75.8% of COL- and TIG-resistant strains) of the isolates are potential biofilm producers; thus, they could be protected from host immunity, antimicrobial agents, disinfectants, and other forms of environmental stress [14,51]. These biofilm-producing isolates could adhere to abiotic and biological surfaces, potentially causing persistent infections in the poultry production environment and negatively impacting economic revenue, and, by extension, to humans, wherein biofilm has been associated with many *E*. *coli* infections, including UTIs [14]. Osman et al. [38] also detected biofilm-producing strains among *E*. *coli* isolates from hatchlings/hatcheries in Egypt. We observed the RDAR, BDAR and PDAR biofilm morphotypes indicating the expression of curli and/or cellulose, and colonization of the DOCs by a diversity of potentially biofilm-forming *E*. *coli* strains. The predominance of the RDAR morphotype in this study (among both COL- and TIG-resistant isolates) is consistent with previous studies [51,77]. However, the majority (69.5%) of the COL-resistant strains and the minority (24.5%) of the TIG-resistant isolates in this work displayed the SAW morphotype, implying they cannot form a biofilm due to the lack of expression of both curli and cellulose [51,77]. Although the RDAR morphotype is speculated to help with biofilm formation at the air–liquid interface [77], none of our isolates are able to form a biofilm at the air–liquid interface, having not formed pellicle which is a specialized structure of connected aerobic bacterial cells surrounded by a matrix of extracellular polymeric substance and localized at the air–liquid interface [78]. This suggests that the pathogenicity of the isolates is somewhat lowered, since the pellicle enables bacterial survival under antibiotic pressure and contributes to longer-than-usual persistence in a hostile environment, such as under drying conditions [78].

The bacterial surface layer (S-layer) is a surface protein that protects against bacteriophages and phagocytosis, confers resistance to low pH, serves as a barrier against lytic enzymes and has adherence characteristics [46]. In this experiment, a sizeable percentage (23.1% and 58.1% of COL- and TIG-resistant strains) featured an S-layer, suggesting that a considerable percentage of the isolates may have the capacity to thwart/survive the host immune response. A moderate proportion (33.7% and 35.5% for COL- and TIG-resistant strains) of isolates in this study expressed cell surface hydrophobicity. Cell surface hydrophobicity (CSH) represents a complex interaction between bacterial surface components and the surrounding environment, playing a crucial role in bacterial adherence and colonization [74,79]. The crystalline surface layer on bacterial organisms contributes to CSH [79], and a positive correlation between cell surface hydrophobicity and biofilm formation has been reported [74].

The significantly higher association observed between the ability to form biofilm, gelatinase activity, haemagglutination, and cell surface hydrophobicity with TIG resistance may suggest that the risk of DOCs distributed in Nigeria being colonized by pathogenic COL-resistant *E*. *coli* is lower than for TIG-resistant *E*. *coli* strains. The absence of caseinase, lecithinase and lipase activities in this study is consistent with previous studies [47], suggesting that these may not be important virulence factors required by *E*. *coli* for the colonization of hatchlings. Nonetheless, previous studies observed the expression of some of these factors in *E*. *coli* isolates from other sources [47,75]. The expression of multiple virulence patterns (19 and 14 for TIG- and COL-resistant isolates) by the isolates in this study indicates a multifaceted virulence profile, posing a concern, as these organisms could potentially cause difficult-to-treat infections in infected individuals.

The limitation of this work was that the lack of assessment of antimicrobial usage in breeder farms hindered the correlation of antimicrobial use with the observed resistance. The number of samples processed may not be large enough to conclude that the prevalence of DOCs colonized by COL- and TIG-resistant *E*. *coli* distributed in the study area is as low or high as observed in this study. Considering there are also lactose non-fermenting *E*. *coli* strains [80], the isolation of only lactose-fermenting *E*. *coli* strains could be an underestimation of the prevalence of DOCs colonized by COL- and TIG-resistant *E*. *coli*. Molecular characterization of the isolates, which would reveal antimicrobial resistance and virulence genes in the isolates, was not performed. Thus, the absence of any assayed phenotypic resistance and/or pathogenic potential by some isolates in this study does not preclude them from being resistant and/or potentially pathogenic.

## 5. Conclusions

This study has demonstrated that a considerable percentage of commercial DOCs distributed to poultry farmers in Nsukka, Southeastern Nigeria, are potential reservoirs of COL- and TIG-resistant *E*. *coli*, with a sizeable percentage of these organisms being potentially pathogenic. The presence of potentially pathogenic COL- and TIG-resistant *E*. *coli* in DOCs, as observed in this study, poses a threat to public and animal health, as these organisms can cause challenging-to-treat poultry diseases and food-borne disease outbreaks, and can jeopardize antimicrobial therapy in infected individuals. The spread of COL- and TIG-resistant *E*. *coli* strains could have a significant impact on the ecology and epidemiology of antimicrobial resistance in Nigeria. Therefore, attention should be paid to the use of antimicrobial agents, including COL and tetracyclines, in livestock husbandry, especially poultry in Nigeria, to mitigate the risk of farm-to-fork transmission along the meat chain, as well as environmental transmission of COL- and TIG-resistant *E*. *coli*.

## Figures and Tables

**Table 1:** Distribution of colistin- and tigecycline-resistant *E*. *coli* in day-old chicks.

Hatchery ID	Number of Birds Sampled	Number (%) of Birds Harbouring *Escherichia coli* Unsusceptible to Antibiotic (N = 250)	
		Colistin	Tigecycline
A	68	31 (45.6)	33 (48.5)
B	88	19 (21.6)	21 (24)
C	30	12 (40)	2 (6.7)
D	25	5 (20)	6 (24)
E	25	15 (60)	0 (0)
F	14	13 (93)	0 (0)
Total	250	95 (38)	62 (24.8)

ID = identity; N = total number of birds sampled.

**Table 2:** Phenotypic virulence patterns of colistin- and tigecycline-resistant *Escherichia coli* from commercial day-old chicks.

S/N	Virulence Pattern	Number (% Frequency) of Isolates	
		Tigecycline-Unsusceptible (n = 56)	Colistin-Unsusceptible (n = 72)
1	Hgl	1 (1.8)	2 (2.8)
2	Sfl	2 (3.6)	10 (13.8)
3	Glt	2 (3.6)	3 (4.2)
4	Csh	3 (5.3)	22 (30.5)
5	Bfm	5 (8.9)	11 (15.2)
6	Bfm-Sfl	5 (8.9)	3 (4.2)
7	Gel-Bfm	4 (7.1)	4 (5.6)
8	Bfm-Csh	2 (3.6)	2 (2.8)
9	Glt-Sfl	1 (1.8)	1 (1.4)
10	Hgl-Glt-Csh	1 (1.8)	3 (4.2)
11	Hgl-Glt-Bfm	2 (3.6)	3 (4.2)
12	Hgl-Bfm-Sfl	5 (8.9)	0 (0.0)
13	Glt-Bfm-Sfl	8 (14.3)	3 (4.2)
14	Glt-Bfm-Csh	2 (3.6)	0 (0.0)
15	Bfm-Sfl-Csh	6 (10.7)	5 (6.9)
16	Gel-Bfm-Sfl-Csh	4 (7.1)	0 (0.0)
17	Hgl-Bfm-Sfl-Csh	2 (3.6)	0 (0.0)
19	Hgl-Glt-Bfm-Sfl-Csh	1 (1.8)	0 (0.0)

Hgl = haemagglutination, Sfl = surface layer, Glt = gelatinase, Bfm = biofilm, Csh = cell surface hydrophobicity.

## Data Availability

The datasets generated and/or analyzed during the current study are available on request through the corresponding author.
